# Claudin expression in pulmonary adenoid cystic carcinoma and mucoepidermoid carcinoma

**DOI:** 10.3389/pore.2023.1611328

**Published:** 2023-08-09

**Authors:** Marton Gyulai, Tunde Harko, Katalin Fabian, Luca Karsko, Laszlo Agocs, Balazs Szigeti, Janos Fillinger, Zoltan Szallasi, Orsolya Pipek, Judit Moldvay

**Affiliations:** ^1^ County Institute of Pulmonology, Torokbalint, Hungary; ^2^ Karoly Racz Doctoral School of Clinical Medicine, Semmelweis University, Budapest, Hungary; ^3^ Department of Pathology, National Koranyi Institute of Pulmonology, Budapest, Hungary; ^4^ Department of Pathology, South-Buda Center Hospital St. Imre University Teaching Hospital, Budapest, Hungary; ^5^ Department of Thoracic Surgery, National Koranyi Institute of Pulmonology, Budapest, Hungary; ^6^ Department of Thoracic Surgery, National Institute of Oncology, Semmelweis University, Budapest, Hungary; ^7^ National Koranyi Institute of Pulmonology, Budapest, Hungary; ^8^ Department of Bioinformatics, Semmelweis University, Budapest, Hungary; ^9^ Computational Health Informatics Program, Boston Children’s Hospital, Harvard Medical School, Boston, MA, United States; ^10^ Danish Cancer Society Research Center, Copenhagen, Denmark; ^11^ Department of Physics of Complex Systems, ELTE Eotvos Lorand University, Budapest, Hungary; ^12^ Ist Department of Pulmonology, National Koranyi Institute of Pulmonology, Budapest, Hungary

**Keywords:** adenoid cystic carcinoma, mucoepidermoid carcinoma, rare lung tumors, claudin expression, immunohistochemistry

## Abstract

**Background:** Although the expression of tight junction protein claudins (CLDNs) is well known in common histological subtypes of lung cancer, it has not been investigated in rare lung cancers. The aim of our study was to examine the expression of different CLDNs in pulmonary salivary gland tumors.

**Methods:** 35 rare lung cancers including pathologically confirmed 12 adenoid cystic carcinomas (ACCs) and 23 mucoepidermoid carcinomas (MECs) were collected retrospectively. Immunohistochemical (IHC) staining was performed on formalin fixed paraffin embedded (FFPE) tumor tissues, and CLDN1, -2, -3, -4, -5, -7, and -18 protein expressions were analyzed. The levels of immunopositivity were determined with H-score. Certain pathological characteristics of ACC and MEC samples (tumor grade, presence of necrosis, presence of blood vessel infiltration, and degree of lymphoid infiltration) were also analyzed.

**Results:** CLDN overexpression was observed in both tumor types, especially in CLDN2, -7, and -18 IHC. Markedly different patterns of CLDN expression were found for ACC and MEC tumors, especially for CLDN1, -2, -4, and -7, although none of these trends remained significant after correction for multiple testing. Positive correlations between expressions of CLDN2 and -5, CLDN3 and -4, and CLDN5 and -18 were also demonstrated. Tumors of never-smokers presented lower levels of CLDN18 than tumors of current smokers (*p*-value: 0.003).

**Conclusion:** This is the first study to comprehensively describe the expression of different CLDNs in lung ACC and MEC. Overexpression of certain CLDNs may pave the way for targeted anti-claudin therapy in these rare histological subtypes of lung cancer.

## Introduction

Adenoid cystic carcinoma (ACC) and mucoepidermoid carcinoma (MEC) belong to the group of rare lung cancers. They originate from the salivary glands of the tracheobronchial tree and represent 0.1%–0.2% of all lung cancer cases [1]. In low-grade and early-stage tumors complete bronchoscopic excision or surgical resection result in long term survival [2]. In high-grade tumors and in metastatic cases, however, the therapeutic options are often limited and combination of radiotherapy and chemotherapy yields only modest survival [3].

Claudins (CLDNs) are important constituents of tight junctions, and play key role in cell proliferation, glandular differentiation, cellular adhesion, and metastasis formation [4]. They serve as a barrier regulating the passage of ions, water and different macromolecules to maintain homeostasis, and work as a fence to keep cell polarity by regulating the separate passages of molecules in the apical and basolateral membrane. At least 27 different CLDN members are known today, all of which are thought to vary in expression depending on location and cell type [5, 6]. Altered expression of different CLDNs was found in a wide variety of human malignancies, and in many tumor types it had prognostic significance [7, 8]. In 2007, our research group was the first to demonstrate that CLDN1, -2, -3, -4, and -7 proteins are expressed in normal bronchial epithelial cells as well as in different histologic subtypes of lung cancer [9]. We observed distinct CLDN mRNA and protein expression profile within the non-small cell lung cancer (NSCLC) group and marked differences between small cell lung cancers (SCLCs) and carcinoids that may have differential diagnostic impact. In our other work, we showed that CLDN expression differences can also be observed between the histological subtypes of lung adenocarcinomas, namely, between tumors with lepidic spread and non-lepidic spread [8]. We also demonstrated the overexpression of different CLDNs, especially CLDN2, -3 and -4, and -7 as has already been described in other tumor types, which might serve as a therapeutic target [10, 11].

Since there is only very little data available on CLDN protein expression in primary bronchial ACC and MEC, the aim of our present work was to comprehensively investigate the CLDN expression in these rare pulmonary cancers in order to determine the potential role of anti-CLDN treatment.

## Patients and methods

### Patients

In our retrospective study, we included 35 pathologically confirmed rare lung tumors including 12 adenoid cystic carcinomas, and 23 mucoepidermoid carcinomas. These patients were diagnosed and treated in the National Koranyi Institute of Pulmonology in Budapest between 1987 and 2023. In 27 cases surgical resection was performed, and eight patients had bronchoscopic tumor excision with rigid bronchoscope under general anesthesia. In the cases of surgical resection pathologic TNM (pTNM), while in the cases of bronchoscopic excision clinical TNM (cTNM) was applied.

The clinicopathological characteristics of patients including age, gender, smoking history, histology, stage, and the type of biopsy material are summarized in Table 1.

**TABLE 1:** Major characteristics of 35 patients diagnosed and treated with adenoid cystic carcinoma and mucoepidermoid carcinoma between 1987 and 2023.

	ACC	MEC	*p*-value
	*N = 12*	*N = 23*	
Gender			1.000^a^
Female	5 (41.7%)	10 (43.5%)	
Male	7 (58.3%)	13 (56.5%)	
Age, years—median (range)	64 (32–81)	48 (19–67)	**0.014** ^b^
Smoking			0.175^a^
Never	4 (33.3%)	11 (47.8%)	
Ex	2 (16.7%)	0 (0.00%)	
Current	4 (33.3%)	7 (30.4%)	
nd	2 (16.7%)	5 (21.7%)	
Grade			**0.010** ^a^
1	0 (0.00%)	11 (47.8%)	
2	10 (83.3%)	9 (39.1%)	
3	2 (16.7%)	3 (13.0%)	
Vascular involvement			0.594^a^
No	10 (83.3%)	21 (91.3%)	
Yes	2 (16.7%)	2 (8.70%)	
Lymphoid infiltration			0.709^a^
Slight	9 (75.0%)	15 (65.2%)	
Moderate	3 (25.0%)	8 (34.8%)	
Necrosis			0.725^a^
No	7 (58.3%)	11 (47.8%)	
Yes	5 (41.7%)	12 (52.2%)	
Stage category			**0.016** ^a^
I	3 (25.0%)	16 (69.6%)	
II	2 (16.7%)	4 (17.4%)	
III	5 (41.7%)	3 (13.0%)	
IV	2 (16.7%)	0 (0.00%)	
Sample type			
Bronchoscopic excision	7 (58.3%)	1 (4.3%)	
Surgical material	5 (41.7%)	22 (95.7%)	

ACC, adenoid cystic carcinoma; MEC, mucoepidermoid carcinoma; nd, no data.

*p*-values are non-adjusted ones, but bold lettering mark those associations that remained significant after correction as well. Adjustment for multiple testing was performed with the Benjamini-Hochberg method.

^a^ Fisher’s exact test.

^b^ Kruskal-Wallis test.

### Histology and TMA construction

Since the vast majority of biopsy tissue samples were obtained before 2020, therefore, all tumor samples were re-examined. The ACC and MEC diagnoses were confirmed with the following immunohistochemical (IHC) markers: pancytokeratin, actin, S100, and p63. Tissue characteristics, such as tumor grade [1–3], presence of necrosis (yes/no), presence of blood vessel infiltration (yes/no), and degree of lymphoid infiltration (slight/moderate) were determined on hematoxylin-eosin stained sections.

In the case of surgical specimens IHC staining was performed on tissue microarrays (TMAs) (5 × 6; diameter, 2 mm) with one or two cores per patient, prepared from selected areas of formalin fixed paraffin embedded (FFPE) tissue samples (TMA Master; 3DHistech, Budapest, Hungary). In the case of bronchoscopic excisions 3-µm-thick sections were prepared for IHC without TMA construction.

### Immunohistochemistry

For immunoreactions, the following antibodies and dilutions were used: CLDN1 mAb (Invitrogen, 1:200), CLDN2 mAb (Invitrogen, 1:300), CLDN3 pAb (Invitrogen, 1:200), CLDN4 mAb (Invitrogen, 1:200), CLDN5 mAb (Invitrogen, 1:20), CLDN7 mAb (Invitrogen, 1:200), and CLDN18 mAb (Invitrogen, 1:200). All primary antibodies were incubated at 42°C for 32 min. IHC staining was performed using the Roche Ventana Benchmark ULTRA automated slide stainer (Ventana Medical Systems, Roche Diagnostics, France) with the UltraView universal DAB IHC Detection Kit (Roche, France). After antibody visualization the slides were counterstained with hematoxylin. As a positive control, CLDN positive lung cancer tissues were used for CLDN1, -2, -3, -4, -5, -7, and -18 reactions.

All sections were examined and evaluated by two experienced pathologists at ×400 magnification. The levels of immunopositivity were determined with H-score, which was calculated by a semi-quantitative assessment of both the intensity of staining (graded as: 0 = non-staining; 1 = weak; 2 = median; or 3 = strong) and the percentage of positive cells (H-score: 0–300). In heterogeneous tumor samples we analyzed the H-score in different regions, and the final score was determined by taking into account the proportion of heterogeneous areas.

### Statistical analysis

All statistical analyses were performed using R version 4.2.1 (R Foundation for Statistical Computing, Vienna, Austria). Categorical and ordinal parameters were statistically analyzed by Fisher’s exact tests. Continuous variables were compared with Mann-Whitney test or Kruskal-Wallis tests. Hierarchical clustering of samples based on the measured expression levels was performed with the ComplexHeatmap R package (v.2.10.0; URL)^1^. The distance matrix was calculated using Euclidean distance measure and the dendrograms were created using the ward.D clustering method. Correlations of expression levels were calculated in a pairwise manner using Pearson correlation. The value of linear correlation coefficient (R) varies from −1 to 1 both values inclusive. Throughout the analyses, the Benjamini–Hochberg correction was used to adjust for multiple testing.

## Results

### Comparison of clinicopathological characteristics of ACC and MEC samples

We observed that ACC tumors tended to have both significantly higher grades (adjusted *p*-value = 0.044, Table 1) and higher stages (adjusted *p*-value = 0.044, Table 1) than MEC tumors. Patients with ACC tumors were also generally older than patients with MEC tumors (medians: 64 vs. 48 years, adjusted *p*-value = 0.044, Table 1). No other significant tendencies could be detected when comparing tumors of the two subtypes regarding either gender, smoking, vascular involvement, lymphoid infiltration or necrosis.

### CLDN immunohistochemistry

**FIGURE 1:**
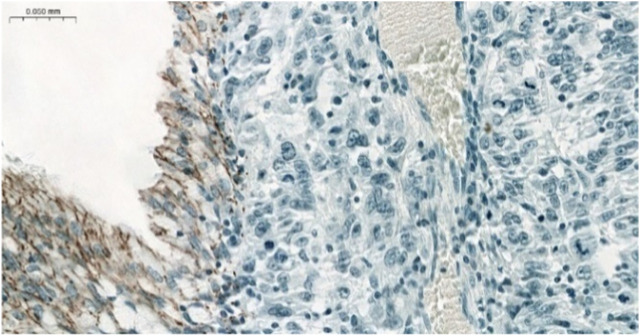
Negative CLDN4 IHC in MEC. Note the positive normal bronchial cells serving as a positive endogenous control (×200).

**FIGURE 2:**
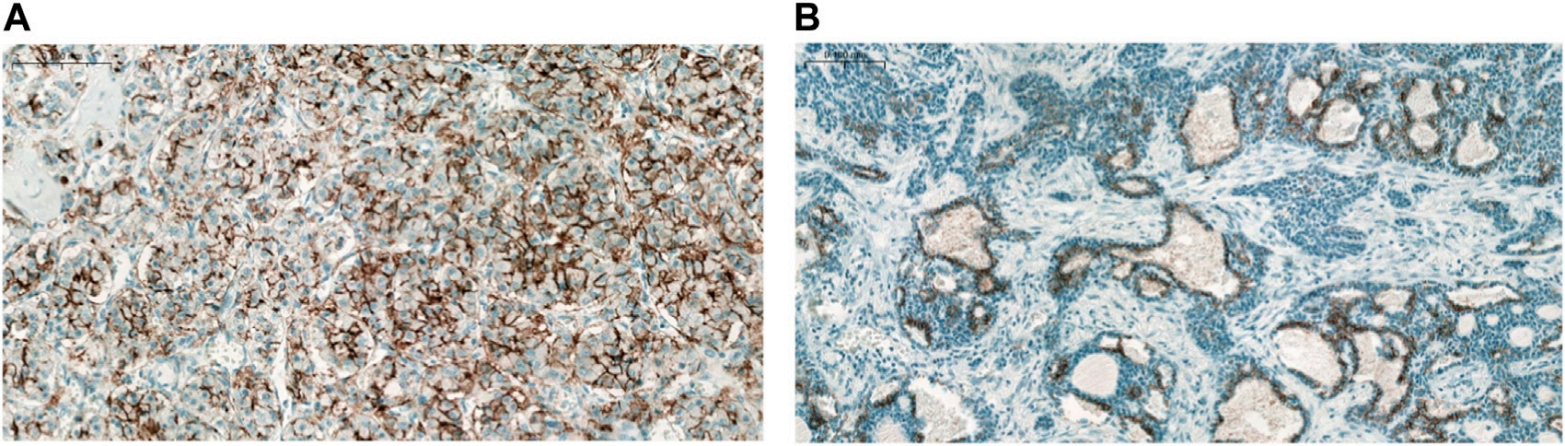
CLDN7 expression in ACC. **(A)** No major intratumoral heterogeneity (×200), **(B)** Marked intratumoral heterogeneity (×100).

**FIGURE 3:**
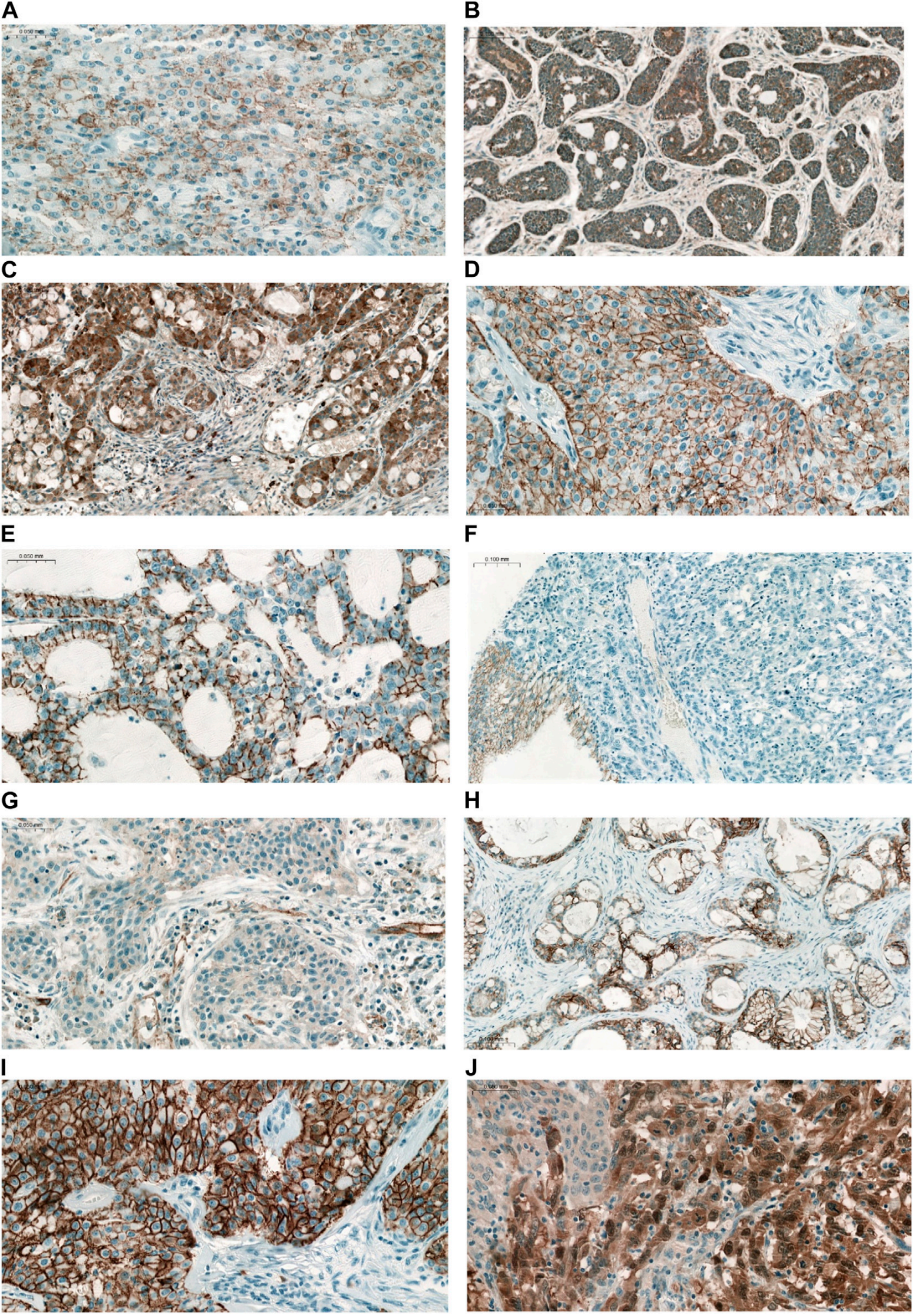
Representative IHC reactions of different CLDNs in pulmonary ACC and MEC. **(A)** CLDN1 MEC (×200), **(B)** CLDN2 ACC (×100), **(C)** CLDN2 MEC (×100), **(D)** CLDN3 MEC (×200), **(E)** CLDN4 ACC (×200), **(F)** CLDN4 MEC (×100). Note the negative tumor cells and the positive normal bronchial cells serving as a positive endogenous control, **(G)** CLDN5 MEC (×200). Weak positive staining in tumor cells, and vessels served as positive endogenous control, **(H)** CLDN7 ACC (×100), **(I)** CLDN7 MEC (×200), **(J)** CLDN18 MEC (×200). Note the heterogeneity of IHC staining.

The majority of CLDN IHC positive tumors showed strong and homogenous staining. In certain cases of CLDN IHC negative tumors, non-tumorous tissue serving as an endogenous positive control helped the correct scoring (Figure 1). Membranous CLDN immunopositivity could be observed in CLDN1, -3, -4, and -7 IHC, while cytoplasmic staining was found in CLDN2, -5, and -18 IHC. Generally, all positive tumors stained diffusely and in fairly uniform manner (Figure 2A). The intensity of immunostaining was usually similar to—or even stronger than—the adjacent normal bronchial epithelium. Heterogeneity of expression in different areas within the same tumor was observed only in very few cases (Figures 2B, 3J). Representative pictures of CLDN IHC in ACC and MEC cases are demonstrated in Figures 3A–J.

#### Associations between CLDN expression and clinicopathological characteristics

Given the low number of available cases, relationships between expression levels and other clinicopathological features were investigated on all data, disregarding tumor subtype.

Out of all measured CLDN expression levels, CLDN4 seemed to be most influenced by different factors. We found that grade 2 tumors expressed higher levels of CLDN4 than grade 1 tumors (median H-scores: 60 vs. 1; Mann-Whitney test *p*-value: 0.045), and a positive correlation could be observed between stage category and CLDN4 expression (R = 0.5, *p*-value: 0.003). CLDN4 levels also seemed to be positively correlated with age (R = 0.49, *p*-value: 0.004).

Additionally, we found that tumors with signs of necrosis expressed higher levels of CLDN5 than tumors without (median H-scores: 60 vs. 0; Mann-Whitney test *p*-value: 0.009).

The most compelling relationship was observed between CLDN18 expression and smoking: tumors of never-smokers presented lower levels of CLDN18 than tumors of current smokers (median H-scores: 70 vs. 190, Mann-Whitney test *p*-value: 0.003).

Out of all these associations, only the latter one remained significant when correcting for multiple testing.

#### Differences in CLDN expression profiles between ACC and MEC

**FIGURE 4:**
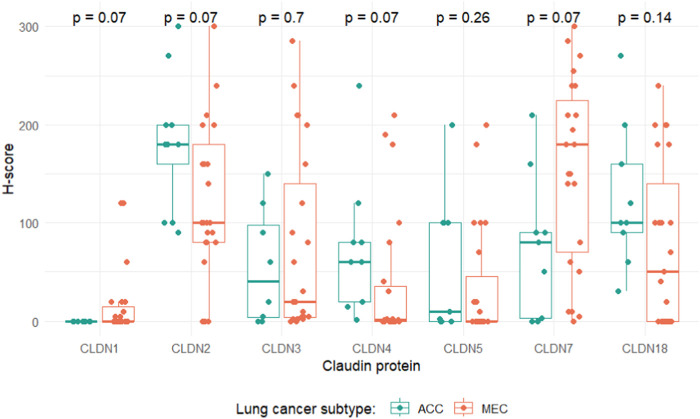
Distributions of claudin expression levels grouped by lung cancer subtype. *p*-values indicate adjusted *p*-values, corrected for multiple testing with the Benjamini-Hochberg method.

Expression levels of CLDN1, -2, -4 and -7 showed markedly different distributions for ACC and MEC tumors. Specifically, ACC samples exhibited a propensity for heightened CLDN2 and -4 expression, whereas MEC cases displayed elevated levels of CLDN1 and -7. However, none of these tendencies remained significant when correcting for multiple testing (Figure 4). In ACC CLDN1 was the only marker that was negative in all tumor tissues, whereas in MEC there was no such a marker that was negative in all samples.

**FIGURE 5:**
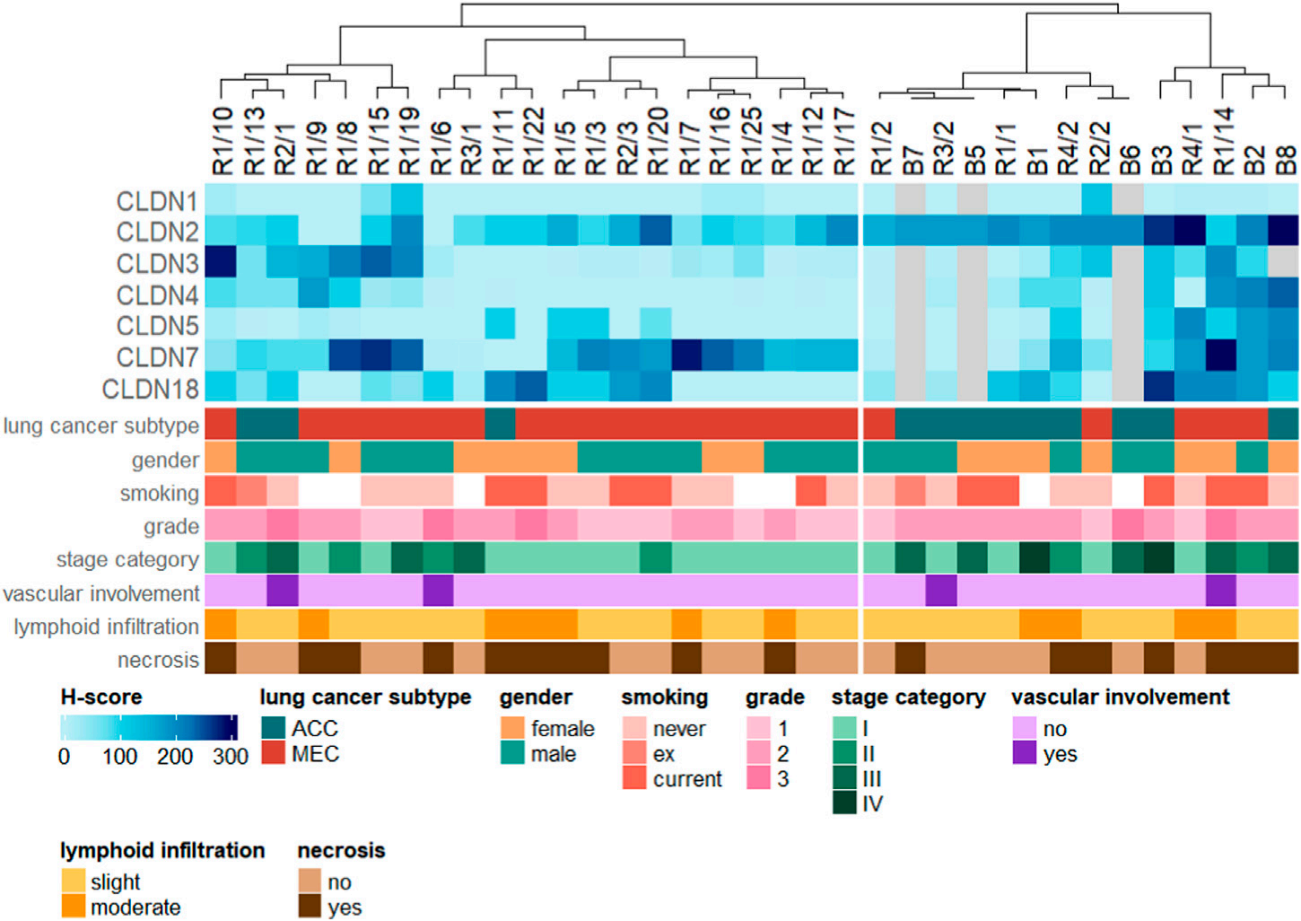
Hierarchical clustering of the samples based on CLDN expression levels. Clinicopathological parameters are shown as column annotations at the bottom.

As an additional insight, a simple unsupervised hierarchical clustering of the samples was performed based on the measured expression levels (Figure 5). The resulting dendrogram of the cases was split to obtain two groups. Column annotations at the bottom of the figure show that lung cancer subtypes within the two groups (with a few exceptions) are fairly uniform, suggesting that patterns of CLDN expression inherently differentiate between ACC and MEC tumors (adjusted *p*-value = 0.032). On the other hand, remaining clinicopathological parameters are relatively well-mixed in the two groups (adjusted *p*-values: 1.0, 1.0, 1.0, 0.468, 1.0, 1.0, 1.0 for gender, smoking, grade, stage category, vascular involvement, lymphoid infiltration and necrosis, respectively), indicating that the main factor influencing the separation based on CLDN expression levels is indeed cancer subtype.

#### Correlations of different CLDN expression levels

Due to the low number of cases, correlations between CLDN expressions were assessed on the whole dataset, regardless of tumor subtype.

**FIGURE 6:**
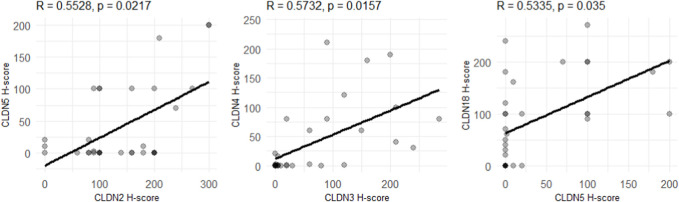
Correlations between CLDN expression levels in the whole dataset. *p*-values indicate adjusted *p*-values, corrected for multiple testing with the Benjamini-Hochberg method. R-values correspond to the Pearson correlation-coefficient.

We identified positive correlations between the expression levels of CLDN5 and -2 (R = 0.55, adjusted *p*-value = 0.02), CLDN3 and -4 (R = 0.57, adjusted *p*-value = 0.02) and CLDN5 and -18 (R = 0.53, adjusted *p*-value = 0.04) (Figure 6). All of these associations remain significant when corrected for multiple testing.

#### FFPE block age-related artifacts

Given the well-known degradation of FFPE blocks over time, we reanalyzed older and newer samples separately to validate the above observed trends. Samples were classified into “old” and “new” cohorts based on the year of FFPE block creation: blocks prepared before 2009 were designated as “old,” while those created thereafter as “new.” The threshold for this categorization was chosen as the median of the FFPE block creation years in our dataset.

Generally, as expected, regarding all CLDN expression levels, old cases were lower expressors than new ones, although in most cases not to a significant extent. Exceptions for this trend were CLDN1 and -4, with very low median H-scores in both sample groups. (Medians and multiple testing-adjusted Kruskal-Wallis test *p*-values for old vs. new cases: 0 vs. 0, *p* = 0.76 (CLDN1), 100 vs. 200, *p* = 0.003 (CLDN2), 20 vs. 50, *p* = 0.90 (CLDN3), 2 vs. 1, *p* = 0.95 (CLDN4), 0 vs. 10, *p* = 0.56 (CLDN5), 140 vs. 150, *p* = 0.76 (CLDN7), 50 vs. 160, *p* = 0.11 (CLDN18).) However, despite these general trends, the results discussed above remain valid for both new and old samples when analyzed separately, albeit in some cases with slightly diminished statistical power.

Regarding the expression patterns of ACC and MEC tumors, the elevated CLDN1 and -7 levels of MEC cases could be observed in both old and new samples. ACC samples were verified to express higher levels of CLDN4 in both cohorts, and CLDN2 in old samples.

## Discussion

The CLDN expression in MEC and ACC is poorly investigated, and the results published so far only refer to head and neck salivary gland tumors [12].

Maria et al. examined CLDN1, - 2, -3, -4, and -5 expression in 10 salivary gland tumor samples (1 parotid and 9 submandibular without further histologic specification) and found that all CLDNs were expressed, except CLDN5 [13]. Aoyama et al. examined CLDN1, -4, and -7 expression in 2 parotid and 2 submandibular salivary gland tumors and described membranous immunopositivity for all studied IHC markers [14]. In our present work we observed CLDN IHC immunopositivity for all studied IHC markers, including CLDN5, however, we found a significant difference in the localization of their immunopositivity and the degree of their expression, as detailed in the description of the results. The observed intertumoral heterogeneity in CLDN expression in ACC seems to be confirmed by recent molecular genetic studies on salivary gland ACC, when molecular genetic differences were described and potentially actionable molecular alterations were found in 28 of 135 studied patients (21%) [15].

If we compare the CLDN expression pattern of ACC with our previously described results in histological subtypes of lung cancer, we find that ACC shows lower expression of CLDN1, -3, and -4, and higher expression of CLDN2 than NSCLC tumors [9]. The CLDN expression pattern of ACC–regarding CLDN1, -2, -3, -4, and -7—is most similar to that of lung adenocarcinoma with lepidic spread [8]. It is worth emphasizing that significant CLDN2 expression was observed in ACC, which is contrary to what we previously observed in SCLC, where there was no CLDN2 IHC positive tumor. Both CLDN5 and CLDN18 were described for the first time in pulmonary ACC. Half of the cases showed moderate CLDN5 immunostaining, while one-third of the tumors showed particularly strong CLDN18 staining.

Regarding CLDN expression in MEC, Aoyama et al. examined CLDN1, -4, and -7 expression in six parotid and three submandibular salivary gland MEC tumors and observed membranous immunostaining in all markers, which is similar to our observations [14]. Aro et al. analyzed CLDN1, -3, -4, and -7 expression in salivary gland MEC [16]. They found that high expression of CLDN1 was seen in low-grade MEC, and it appeared to be a marker of good prognosis. High CLDN3 expression was seen in intermediate- and high-grade MEC, while it was low in low-grade MEC. In our work grading was only observed to have an association with the expression of CLDN4 (with grade 2 tumors expressing higher levels than grade 1 tumors), but this trend was neither specific to MEC cases, nor remained significant after multiple testing correction.

In our own study, the most prominent CLDN positivity in MEC was observed for CLDN2 and CLDN7, although IHC negative tumors were also found for both markers.

If we compare the CLDN expression pattern of MEC with our previously described results in histological subtypes of lung cancer, we could observe high CLDN2 and CLDN7 expression, as well as low CLDN3 expression, which was most characteristic of squamous cell carcinoma among lung cancers [9].

It is undeniable that our results might suffer from the limitations of having a relatively small number of cases. Undoubtedly, a significantly larger cohort would give more reliable insights into the expression of different CLDNs in pulmonary ACC and MEC, however, in rare tumors, usually it takes many years to collect the number of cases suitable for such examinations. Furthermore, it will be necessary to expand the range of tested anti-CLDN antibodies (e.g., CLDN6 and -18.2) and a wider clinical data analysis. Other limitation might be the age of FFPE tissue samples, which may affect IHC reactions. In order to investigate this, we compared the IHC results of old and new tissue samples and observed that, as expected, measured H-scores are generally lower for old cases. However, we also validated our results obtained on the whole dataset separately on old and new samples and found that the previously established associations between various parameters hold true for both cohorts, although naturally with lower statistical power. Notwithstanding this encouraging outcome, the possibility of age-related FFPE block artifacts highlights the necessity of international, multi-center studies that could provide an increased sample size even for rare malignancies with a focus on analyzing relatively recent tissue samples.

In rare pulmonary tumors, there is unmet need to develop more efficient therapeutic modalities, especially for patients with advanced-stage disease [17–22].

In a recent study of Roehlen et al. targeting non-junctional CLDN1 (on the basolateral membrane of the human hepatocyte) markedly suppressed tumor growth and invasion in cell line-based models of hepatocellular carcinoma and patient-derived 3D *ex vivo* models. Moreover, the robust effect on tumor growth was confirmed *in vivo* in a large series of cell line-derived xenograft and patient-derived xenograft mouse models [23]. In our work ACC did not express CLDN1, and MEC showed only mild expression, therefore targeting CLDN1 is not a promising therapeutic strategy.

On the contrary, CLDN2 expression was very characteristic for both ACC and MEC. Wei et al. demonstrated in colorectal cancer that CLDN2 is upregulated and associated with poor survival, and CLDN2 depletion significantly promoted N-myc downstream-regulated gene 1 (NDRG1) transcription, leading to termination of the cancer cell growth and metastasis *in vitro* and *in vivo* [24]. Thus, this study suggested that CLDN2 may serve as a promising target for colorectal cancer treatment.

*Clostridium perfringens* enterotoxin (CPE) triggers lysis of epithelial cells through binding to tight-junction proteins CLDN3 and CLDN4, therefore, high expression of these proteins might facilitate CLDN-targeted therapy using CPE. Maeda et al. investigated the therapeutic effect of CPE in prostate cancer xenografts in athymic mice [25]. Reduced expression of CLDN4, but not CLDN3, led to remarkable decreases of cytotoxicity, and the injection of CPE around PC3 xenografts significantly suppressed tumor growth. It has been published by Luo et al. that targeting CLDN-4 enhances chemosensitivity in breast cancer [26]. In our study, both ACC and MEC showed CLDN3 and -4 IHC positivity, but only to a moderate extent on average. Certain tumor samples, however, especially in the MEC group showed high expression of CLDN3 and -4.

Until now, no therapeutic approach against CLDN5 has been described. In our work the IHC scores of CLDN5 both in ACC and MEC varied from 0-200, therefore, in the case of some highly expressing tumors, anti-CLDN5 treatment can theoretically be considered.

CLDN7 is highly expressed both in the lung cancers we previously investigated as well as in the ACC and MEC cases we studied now, especially in half of the MEC tumors. The role of CLDN7 in carcinogenesis is somewhat controversial. In 2018, Li et al. demonstrated a previously undescribed role of CLDN7 as a clear cell renal cell carcinoma suppressor and suggested that loss of CLDN7 potentiates epithelial-mesenchymal transition and tumor progression [27]. On the contrary, Philip et al. reported that CLDN7 promotes the epithelial-mesenchymal transition in human colorectal cancer cell lines [28]. In an interesting study by Hoggard et al. CLDN7 was found to increase chemosensitivity to cisplatin through the upregulation of caspase pathway in human NCI-H522 lung cancer cells [29].

Overexpression of CLDN18 paves the way for anti-CLDN18 antibody therapy. Xu et al. published the results of the application of a novel anti-CLDN18.2/anti-CD3 bispecific antibody, which exhibited low affinity for anti-CD3, highly specific binding, potent cytotoxicity, and anti-tumor activity [30]. Recently, Wang et al. reported that CLDN18 isoform 2 (CLDN18.2) is highly expressed in primary ovarian mucinous carcinomas and metastatic gastrointestinal mucinous carcinomas derived from upper gastrointestinal tract primary tumors [31]. They concluded that CLDN18.2-targeted therapy might serve as a potential therapeutic strategy for primary ovarian mucinous carcinomas and metastatic gastrointestinal mucinous carcinomas from the upper gastrointestinal tract. Very recently, in a multicenter, randomized, double-blind, phase 3 trial (SPOTLIGHT), zolbetuximab, a monoclonal antibody targeting CLDN18.2 was found to significantly prolong progression-free survival and overall survival when combined with mFOLFOX6 versus placebo plus mFOLFOX6 in patients with CLDN18.2-positive, HER2-negative, locally advanced unresectable or metastatic gastric or gastro-oesophageal junction adenocarcinoma [32]. The results of this clinical trial may bring a paradigm shift in the treatment of malignant tumors that overexpress CLDN proteins. In our cohort CLDN18 overexpression was pronounced in 2/3 of the ACC and in half of the MEC cases. The planned CLDN18.2 immunostainings could not be performed due to technical reasons.

In summary, this is the first study which comprehensively describes the CLDN expression pattern in pulmonary ACC and MEC. The overexpression of certain CLDNs both in ACC and MEC opens the door to anti-claudin treatment in these rare lung cancer subtypes. Based on the results of our present study, in the case of pulmonary ACC and MEC, oncotherapies against CLDN-2, -7, and -18 arise mostly, but in some cases CLDN-3 and -4 overexpression might also serve as a target.

## Data Availability

The original contributions presented in the study are included in the article/supplementary material, further inquiries can be directed to the corresponding author.

## Footnotes

1
https://jokergoo.github.io/ComplexHeatmap-reference/book/; date last accessed: 5/10/2021.

## Abbreviations

ACC, adenoid cystic carcinoma; CLDN, claudin; IHC, immunohistochemistry; MEC, mucoepidermoid carcinoma; NSCLC, non-small cell lung cancer; SCLC, small cell lung cancer.
